# Re-expression of the p53 Gene by Inhibiting the Mdm-2 Receptor in Wild-type p53 Tumors for the Treatment of Glioblastoma: A Mini Review

**DOI:** 10.7759/cureus.3034

**Published:** 2018-07-23

**Authors:** Syed Ijlal Ahmed, Syeda Beenish Bareeqa, Syeda Sana Samar

**Affiliations:** 1 Graduate, Liaquat National Hospital and Medical College, Karachi, PAK; 2 Medical Student, Jinnah Medical and Dental College, Riyadh, PAK; 3 Medical Student, Jinnah Sindh Medical University, Karachi , PAK

**Keywords:** mdm-2 antagonist, glioblastoma (gbm), p53 gene

## Abstract

Glioblastoma multiforme (GBM) has been the topic of immense research in recent years. The suitable therapeutic approach towards this wild-type p53 tumor has been the topic of ongoing discussion for some decades now. There has been a substantial debate about the role of mouse double minute-2 (MDM-2) antagonists in the treatment of GBM recently. We have reviewed the current data in our study to establish the consensus about recent interventions. Our review of the literature suggests that MDM-2 antagonists are currently a more suitable approach with improved efficacy, and it might be utilized in the future for significant results. Newer analogs of MDM-2 antagonists with better pharmacokinetics profiles and the least drug-drug interactions were also discussed in our research. It was concluded that MDM-2 antagonists are improved therapy against GBM but evidential proof with more experimental studies is needed to standardize this therapy in near future.

## Introduction and background

Mutation in the genetic makeup of humans is a paramount event in the evolution of cancer. Tumor suppressor gene p53, also regarded as the “guardian of the genome,” is the most frequent participant in neoplastic pathogenesis. Mouse double minute-2 (MDM-2) also known as E3 ubiquitin-protein ligase, is a protein that performs an essential role in the negative regulation of p53 tumor suppressor gene. The MDM-2 gene does the encoding of a relative protein and is considered an oncogene. The aberrant function of Tp53 is the single-most common factor in glioblastoma. The MDM-2 oncogene inactivates p53 by directly inhibiting the expression and activity of Tp53.

## Review

Inhibition of p53 activity by the MDM-2 protein results due to the following three mechanisms: 1) acting as an E3 ubiquitin ligase, which induces p53 degradation; 2) the binding and blockage of the p53 transcriptional activation domain, and 3) exporting p53 to an extranuclear compartment. Small-molecule antagonists of MDM-2 hinder the p53-MDM2 interactions and initiate p53 signaling in the glioblastoma cell. The use of novel peptides to block the MDM-2 pathway is the most commonly utilized molecule to fulfill this purpose.

Literature search strategy

A literature search was conducted using keywords like ‘MDM-2 antagonist,’ ‘Glioblastoma multiforme (GBM),’ and ‘p53 gene.’ It was conducted on Ovid Medline, PubMed, and Google Scholar. The literature search was limited to the articles published from 2011 up to the present. Only English-language literature was included in this review.

Inclusion and exclusion criteria

Review of case reports, case series, cohorts, and randomized controlled trials were included in this research; however, review articles, dissertations, and cross-sectional surveys were excluded. Studies in non-English language literature, studies with incomplete data, and studies with unavailable full texts were also excluded from this review. A total of nine articles were included in this study on the basis of inclusion/exclusion criteria.

A study by Costa et al. reported the use of novel palmitoylated D-peptide in wild-type GBM. This therapy has greater affinity and specificity for wild-type p53 tumor cells, but its poor central nervous system (CNS) penetration hinders the delivery of peptide molecules to target cells. For this purpose, liposome was chosen as a carrier for D-peptide transport to the targeted share. This resulted in an effective antagonism for MDM-2 molecules [1].

Verreault et al. conducted a study that showed that MDM-2 amplified patient-derived GBM cell lines (PDCL) had a 44 times more sensitivity to the RG7112 MDM-2 antagonist than non-MDM2 amplified models. The measuring criteria for that were the reduction in the growth pattern of a tumor, increased cytotoxicity for tumor cells, and increment in survival rate. More importantly, the pharmacokinetics profile of RG7112 MDM-2 inhibitor in mice supported the fact that it can easily cross the blood-brain barrier, as most of the MDM-2 antagonist experimented previously were unable to cross the blood-brain barrier [2].

 A recent study by Chen et al. also signifies the importance of cyclic RGD conjugated peptide micelle (RGD-M) in the allowance of a stapled peptide antagonist of both MDM2 and MDMX (sPMI) into the CNS. RGD-M/sPMI exhibits potent activity in the suppression of GBM in the nude mice xenograft model. It also signifies the enhanced efficacy of RGD-M/sPMI for temozolomide - a standard chemotherapy drug for GBM [3].

A study conducted by Villalonga-Planells et al. provided an evidential proof of cellular senescence in GBM cell lineages after treating the cultured tissue with nutlin-3a (small molecule MDM-2 antagonist). Primarily cultured samples were obtained from nine patients of Hospital de Bellvitge. Patients were diagnosed as cases of grade IV gliomas and the tissue obtained was cultured within an hour after resection. It was also reported that nutlin-3a mediated senescence was markedly dependent on functional p53 machinery. GBM cells with an impaired p53 pathway were found to be insensitive to nutlin-3a therapy [4].

Schneider et al., in 2016, highlighted the significance of archazolid, a drug that inhibits the activity of V-ATPase (a multisubunit proton pump) to increase the levels of p53 protein in cancer cells. In this study, an evidential proof of synergism among archazolid with the MDM-2 antagonist (nutlin 2a) in human GBM cell lines (U87MG) was observed. It was inferred that the efficiency of re-expression of gene p53 with adjuvant therapy is far more promising than with a single drug regimen, and it is considered to be a much better strategy in treating the wild-type of p53 tumors [5].

A study by Daniele et al. tested the mechanism of ISA27 (analogs of spirooxoindolepyrrolidine) in immune-compromised nude mice incarnate with GBM xenograft, which exhibited a dose-dependent inhibitory effect on wild-type p53 cell lineages. Further reduction in cellular viability of GBM was associated with the synergistic effect of ISA27 with temozolomide (TMZ), a standard anti-GBM therapy. This results in a reduction of the dosage and adverse effects of TMZ [6].

Another study by Daniele et al. clarified the idea of combination therapy for hyperactivated AKT/mTOR and MDM2 inhibition to reboot the p53 functionality. The human U87MG cell line was obtained from the National Institute for Cancer Research of Genoa (Italy). It was first cultured and then treated with FC85 and ISA27. Both compounds induced time- and concentration-dependent apoptosis in GBM stem cells. This study is supporting the fact that two pathways interact conjointly in the activation of the p53 tumor suppressor gene pathway [7].

A similar study conducted by Katrin et al. in 2014 highlighted the significance of the PI3K/AKT/mTOR pathway in improving the survival of patients with high-grade gliomas. In this cohort study, samples from 103 patients with low- and high-grade gliomas operated at the University Hospital, Zurich, Switzerland, were considered as cases while four samples of normal brain tissue collected by autopsy were derived as a control. Western blot analysis of apoptosis markers indicated the reduction in cell proliferation and the induction of the apoptotic pathway in GSCs [8].

Sato et al. suggested that the regulation of the MEK-ERK-MDM-2-p53 pathway plays a promising role in improving the therapeutic efficacy of temozolomide against GBM. Tumor samples for stem-like GBM cells culture were derived from patients. Thus, it was concluded that MEK/ERK/MDM-2 inhibition promotes the commitment of stem-like glioblastoma cells to differentiation and, thereby, minimizes their malignant potential [9].

## Conclusions

From the review of the evidence, we concluded that MDM-2 antagonist therapy showed improved efficacy towards GBM. It can be utilized as a new target molecule for the treatment of GBM but further experimental studies need to be conducted before standardizing this therapy as a suitable approach to GBM.
